# CD137 Regulates Bone Loss *via* the p53 Wnt/β-Catenin Signaling Pathways in Aged Mice

**DOI:** 10.3389/fendo.2022.922501

**Published:** 2022-06-30

**Authors:** Jiyu Han, Yanhong Wang, Haichao Zhou, Yingqi Zhang, Daqian Wan

**Affiliations:** ^1^ Department of Orthopedics, Tongji Hospital, School of Medicine, Tongji University, Shanghai, China; ^2^ Key Laboratory of Spine and Spinal Cord Injury Repair and Regeneration, Ministry of Education, Shanghai, China

**Keywords:** osteoporosis, Bioinformatics, gene expression, hub genes, CD 137

## Abstract

Senile osteoporosis is a chronic skeletal disease, leading to increased bone brittleness and risk of fragile fractures. With the acceleration of population aging, osteoporosis has gradually become one of the most serious and prevalent problems worldwide. Bone formation is highly dependent on the proper osteogenic differentiation of bone marrow mesenchymal stem cells (BMSCs) in the bone marrow microenvironment, which is generated by the functional relationship among different cell types, including osteoblasts, adipogenic cells, and bone marrow stromal cells in the bone marrow. It is still not clear how osteoporosis is caused by its molecular mechanism. With aging, bone marrow is able to restrain osteogenesis. Discovering the underlying signals that oppose BMSC osteogenic differentiation from the bone marrow microenvironment and identifying the unusual changes in BMSCs with aging is important to elucidate possible mechanisms of senile osteoporosis. We used 3 gene expression profiles (GSE35956, GSE35957, and GSE35959) associated with osteoporosis. And a protein-protein interaction (PPI) network was also built to identify the promising gene CD137. After that, we performed *in vivo* experiments to verify its function and mechanism. In this experiment, we found that significant bone loss was observed in aged (18-month-old) mice compared with young (6-month-old) mice. The adipose tissue in bone marrow cavity from aged mice reached above 10 times more than young mice. Combining bioinformatics analysis and vivo experiments, we inferred that CD137 might be involved in the p53 and canonical Wnt/β-catenin signaling pathways and thereby influenced bone mass through regulation of marrow adipogenesis. Importantly, osteoporosis can be rescued by blocking CD137 signaling *in vivo*. Our research will contribute to our understanding not only of the pathogenesis of age-related bone loss but also to the identification of new targets for treating senile osteoporosis.

## Introduction

Nowadays, osteoporosis has become a major public health problem worldwide (1), with more than 200 million people suffering from osteoporosis. Primary osteoporosis can be divided into two major categories: postmenopausal osteoporosis, which stems from estrogen deprivation (2), and senile osteoporosis. In line with population aging, the number of senile osteoporosis will also increase (3). The incidence of senile osteoporosis has shown to increase with advancing age. Senile osteoporosis is a health problem that commonly affects the elderly with an increased risk of hip fracture (4, 5). Its action mechanism is that osteoporosis alters the trabecular microstructure of cancellous bone, which increases the fracture risk. There are two types of bone structure in the skeleton: external cortical bone and internal cancellous bone. The “trabecular” or trabeculae bone is the primary anatomical and functional part of internal cancellous bone. There is a highly porous network of struts and rods in the trabecular bone that houses bone marrow (6). During the elderly bone turnover, bone marrow elements represent the most important factor which influences the microscopic structure of cancellous trabecular bone.

The bone homeostasis of microscopic structure in bone marrow is finely maintained by a harmony of osteogenesis (bone formation) and osteoclastogenesis (bone resorption) (7). In the bone marrow, osteoblasts and osteoclasts are both derived from bone marrow stromal cells (BMSCs) (8). There have been dozens of studies showing that indicate that inhibiting the osteoclast differentiation and promoting the osteogenic differentiation of BMSCs can help delay the development of osteoporosis (9). Because BMSCs exhibit a multipotent differentiation capacity, the relationship between osteogenic and adipogenic lineage differentiation of BMSCs is mutually inhibitory (10). So investigating the mechanisms of promoting osteogenesis and suppressing adipogenic differentiation is critical for senile osteoporosis.

As is well known, BMSCs are unspecialized and multipotential cells related to senile osteoporosis.BMSCs are located in specific microenvironments within the bone marrow, which modulate their proliferation, differentiation, and population (11). The bone marrow microenvironment, a versatile dynamic system with an extraordinarily complex and heterogeneous environment, is generated by the interaction of different types of bone marrow cells *via* topically produced soluble factors that have an autocrine, paracrine, and endocrine function (11, 12). It plays a critical role for osteogenesis and bone homeostasis in the context of the physiological bone marrow microenvironment. In such a case, BMSCs could inhibit osteoclast and adipocyte differentiation by a series of events. Meanwhile, they could also enhance osteoblast differentiation (13). It is known, however, that the bone marrow microenvironment changes significantly and provides signals that repress osteogenesis with increasing age (14). Finding the signals that inhibit BMSCs osteoblast differentiation and identifying the changes that appear in BMSCs with aging are a crucial step toward elucidating the molecular mechanisms of senile osteoporosis.

As high-throughput screening techniques evolve, RNA microarray analysis is becoming a revolutionary tool for identifying differentially expressed genes (DEGs). Public, large-scale gene expression data sets, such as Gene Expression Omnibus (GEO), have made it possible to identify a wide range of disease biomarkers (15). A number of studies have investigated the molecular mechanisms and hub genes of senile osteoporosis by bioinformatics technology (16). Based on bioinformatics analysis and a previous study (17), we found that the CD137 gene was a crucial gene for senile osteoporosis and was associated with adipogenic-related signaling pathways.

The tumor necrosis factor receptor (TNFR) superfamily member CD137 (also known as 4-1BB or TNFRSF9) was identified as an inducible costimulatory molecule for activated T cells and innate lymphoid cells. There is only one known ligand of CD137 (TNFRSF9), and that is CD137-Ligand (CD137L), which is expressed on macrophages, activated B cells, and dendritic cells. At present, CD137 receptor agonists-targeted therapeutics are rapidly being investigated and developed to promote antitumor T-cell responses in cancer immunotherapy.

Nevertheless, very few studies have looked at CD137 role in the senile osteoporosis, and these researches only examine how CD137 affects osteoclast formation and function. Thus, CD137 was selected for further verification and study. By using vivo experiments in mice, CD137 influenced bone mass through the regulation of marrow adipogenesis. In addition, blocking CD137 signaling could rescue the development of osteoporosis. Researchers hope that our study will contribute to a better understanding of bone loss associated with aging and find out better therapeutic options for senile osteoporosis.

## Materials and Methods

### Microarray Dataset Selection

The present study selected GSE35956, GSE35957, and GSE35959 gene expression profiling data (18), which were never systemically studied, from the GEO database. Essential information of the included datasets was shown in **Table 1**. This study wasn’t conducted on human tissue specimens, and 3 sets of microarray data were downloaded from GEO. Due to this, consistent with any current decree in China, the analysis didn’t need associate Institutional ethical review board or Human Research Ethics Committee approval or patient consent.

**Table 1:** Basic information of the microarray datasets.

ID	Platform	Data type	Author	update date	Country	Sample type	n (normal)	n (senile osteoporosis)
GSE35956	GPL570	mRNA	Peggy Benisch et	Mar 25, 2019	Germany	Human tissues	5	5
GSE35957	GPL570	mRNA	Peggy Benisch et	Mar 25, 2019	Germany	Human tissues	5	5
GSE35959	GPL570	mRNA	Peggy Benisch et	Mar 25, 2019	Germany	Human tissues	9	10

### Identification of DEGs

GEO2R (http://www.ncbi.nlm.nih.gov/geo/geo2r) is a Web-based (WEB) interactive instrument with R (R Core Team, version 3.6.3) and is used to identify DEGs between osteoporotic patient’s bone marrow and nonosteoporotic donor’s bone marrow. Based on the statistical significance threshold of adj. p-value < 0.05 and |log_2_ fold‐change| > 1, the significant DEGs between groups were screened. Volcano plots were made by using R language and packages ggplot2 in R and Venn diagrams were generated by using the same tool. Next, to help us better understand DEGs, correlation analysis was applied and conducted by using Spearman correlation analysis and visualized by adopting the ggplot2 package. Lastly, the ggplot2 package was employed to create the heatmap.

### Analysis of DEGs Concerning GO and KEGG Pathway Enrichment

The Gene Ontology (GO) term analysis consists of three parts: Cell component (CC), biological process (BP), and molecular function (MF), while gene functions and enriched pathways can be analyzed with the Kyoto Encyclopedia of Genes and Genomes (KEGG) database. DEGs enrichment analysis using GO enrichment analysis and KEGG pathway analysis was conducted by the clusterProfiler (19) R package. Afterward, we used the org.Hs.eg.db (version 3.4.0) and GOplot R (version 1.0.2) packages for analysis and visualization of the results by generating cluster plots (20). A GO term analysis and a KEGG pathway analysis were carried out for DEGs (p-value cutoff =0.05, q value cutoff =0.2).

### Construction of the Predicted PPI Network and Hub Gene Identification

STRING(https://string-db.org/), a well-known online biological tool for the prediction of Protein-protein interaction (PPI), comprises direct (physical) and indirect (functional) associations (21). With the help of version 11.0 of the PPI database STRING, we identified the DEGs involved in the PPI. In this PPI network, the required interaction score for determining a significant interplay was medium confidence (0.400) as the cut-off criteria. Second, the PPI network was visualization with Cytoscape software (22) (version 3.8.2). Finally, the plug-in cytoHubba was used to find out the hub genes among the screened DEGs. Through the use of cytoHubba, we selected hub genes based on the top 5 nodes ranked by degree.

### Animals

A group of male C57BL/6 mice was housed under SPF conditions (SIPPR-BK Laboratory Animal Co.Ltd, Shanghai, China). We obtained approval for all animal operations from the Animal Ethics Committee of Tongji Hospital affiliated with Tongji University School of Medicine.

A total of 10-12 animals were included in each group. Anesthesia was administered with isoflurane, followed by cervical dislocation, before the animals were euthanized. A micro-computed tomography (micro-CT) scan of the right tibia was performed. The left femurs separated from mice were fixed with paraformaldehyde, then frozen sections were performed after decalcification.

Animals received 100 µg/animal per week of neutralization anti-CD137 mAb (BioLegend, San Diego, CA, USA) intraperitoneally in 0.9% saline. In the eighth week, the animals were euthanized for micro-CT and morphometric analysis.

### Micro-CT

An ethanol solution of 70% was used for preserving tibiae. A micro-CT scanner (SkyScan 1172; Bruker-micro-CT, Kontich, Belgium) was used to scan the bones. The American Society for Bone and Mineral Research recommended standard nomenclature and guidelines for assessment of bone microstructure (23).

A scan of the cortex was taken at the midpoints of the tibias with an isotropic pixel size of 21 μm and a slice thickness of 21 μm. Using this data, the average cross-sectional area (mm^2^), bone area (mm^2^), and cortical bone thickness (Ct.Th) can be calculated. An energy level of 55 kVp, intensity of 145 μA, and a fixed threshold of 220 were used for the scan of the trabecular bones of the proximal tibia. In the secondary spongiosa where the growth plate is 0.6mm proximal and extends 1.5mm distally, we assessed the bone volume fraction and microarchitecture. We made 230 consecutive slices along the growth plate and extended in a distal direction, and we selected 100 contiguous slices for analysis. In this study, the key bone parameter was BV/TV (the ratio of calcified to uncalcified tissue in the selected area of interest), TbN (trabecular number), and TbSp (trabecular bone separation).

### Oil Red O Staining

The decalcified and paraffin-embedded fixed tissues were followed by a micro-CT scan. Mounting serial sections of embedded specimens to slides, deparaffinizing and rehydrating them in a graduated manner were performed. After decalcification, frozen sections of the specimens were cut and stained with Oil Red O.

### Statistical Analysis

Bioinformatic data analyses were conducted by R statistical software and p-values < 0.05 were regarded to be statistically significant. Student’s t-test was used to calculate statistical significance for two-sample comparisons. SPSS 16.0 was used to perform the ANOVA for multiple comparisons. When ANOVA is used to find significant differences, Tukey’s test is used. The Spearman correlation analysis was used to determine the relationship between CD137 and CD137L levels and trabecular bone volume fraction. The significance level was set at p < 0.05. Selected data were obtained from three independent experiments. Unless otherwise stated, all figures are the mean ± SEM.

## Results

### Identification of DEGs in CD137

**Figure 1:**
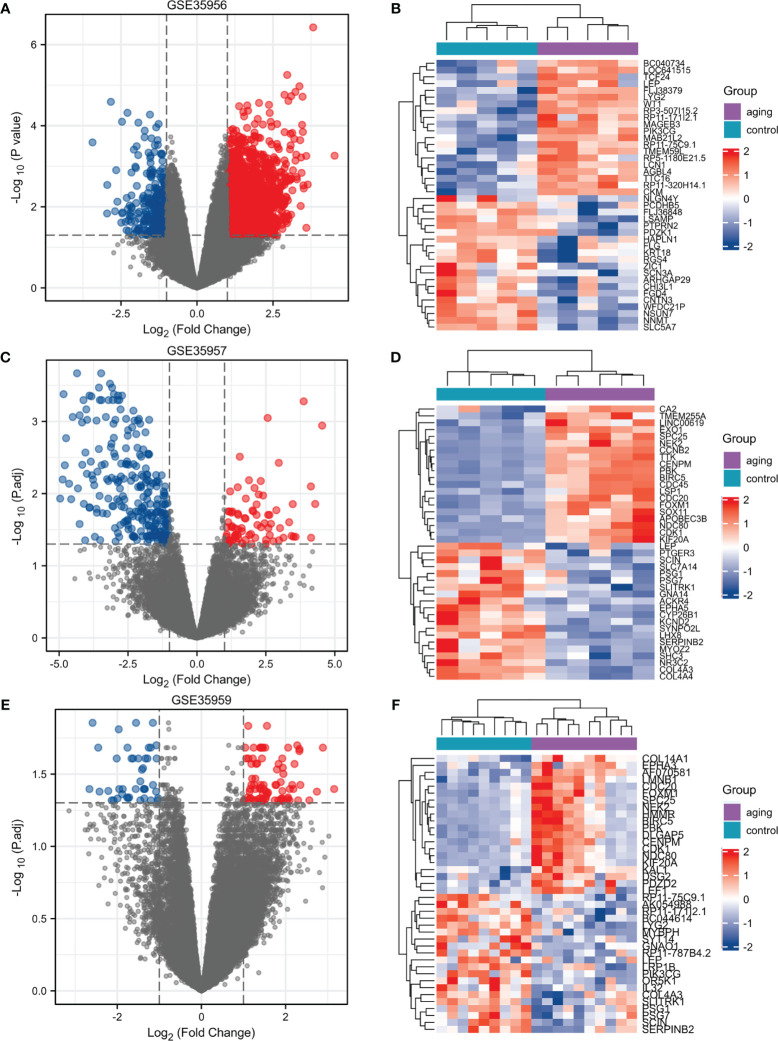
Identification of DEGs in osteoporosis patients and non-osteoporotic donors. **(A–F)** Three volcano plots and Heat maps show all the expressed genes from GSE35956, GSE35957 and GSE35959. Blue and red in **(A–F)** respectively represent the down and up regulated genes. Each column represents a probe, and each row represents a gene. DEGs, differentially expressed genes.

We selected three microarray gene expression datasets (GSE35956, GSE35957, and GSE35959) associated with osteoporosis from the GEO database. By comparing an osteoporotic patient’s bone marrow with that of a nonosteoporotic donor, we performed gene differential analysis. Based on the identification of the microarray results of the GSE35956 datasets, 1664 up-regulated and 267 down-regulated genes were identified in GSE35956. 1788 DEGs were identified in the GSE35957 dataset (827 upregulated, 961 downregulated), while 1683 were identified in the GSE35959 dataset (1315 upregulated, 368 downregulated). The volcano plots of each dataset are depicted for the visualization of DEGs in **Figures 1A, C, E**. When plotting volcanoes, blue indicates downregulation while red indicates upregulation. And the top 20 up-or down-regulated DEGs were selected for Spearman correlation analysis. **Figures lB, D, F** showed a heatmap of the correlation analysis between the DEGs. To study the genetic basis of biological processes, studying DEGs has become crucial.

### GO and KEGG Enrichment Analysis of DEGs

**Figure 2:**
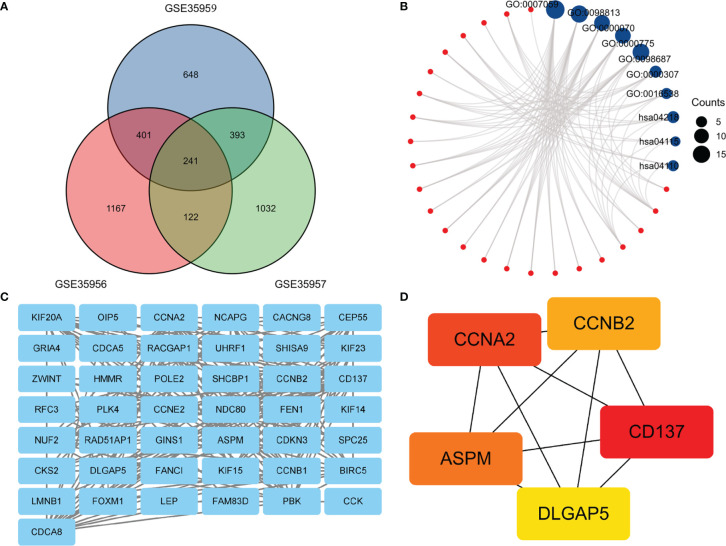
**(A)** An extended range of DEGs were selected from GSE35956, GSE35957 and GSE35959. |log FC| > 1, P value < 0.05. **(B)**. GO and KEGG enrichment analysis of DEGs. **(C)** PPI network construction and analysis of hub genes. **(D)** Hub genes were selected according to the top 5 nodes ranked by degree using cyto-Hubba. DEGs, differentially expressed genes; KEGG, Kyoto Encyclopedia of Genes and Genomes; PPI, protein–protein interaction.

In this heatmap, blue indicated down-regulation, while red indicated up-regulation. The Venn diagram showed a total of 241 overlapping DEGs in **Figure 2A**. Then we analyzed overlapping DEGs based on GO and KEGG pathway enrichment. Based on the GO analysis, 241 overlapping DEGs were enriched for 126 BP terms, 41CC terms, and 1 MF term. Under BP terms (**Figure 2B**), DEGs were primarily enriched in chromosome segregation, nuclear chromosome segregation, and mitotic sister chromatid segregation. For CC terms (**Figure 2B**), DEGs were primarily enriched in chromosome, centromeric region, chromosomal region, and cyclin-dependent protein kinase holoenzyme complex. It was revealed from Enrichment analysis of MF terms (**Figure 2B**) that most DEGs were enriched in cyclin-dependent protein serine/threonine kinase regulator activity. The enrichment analysis of KEGG pathways (**Figure 2B**) included 3 KEGG pathways, and most of the DEGs were enriched significantly in the Cellular senescence, p53 signaling pathway, and Cell cycle. The relationship between DEGs and KEGG pathways was exhibited in **Figure 2B**. Mechanisms behind senile osteoporosis may be closely related to the enrichment of GO or KEGG.

### PPI Network Construction and Analysis of Hub Genes

In the PPI network, a total of 241 DEGs were included, which originated from the STRING database. The construction of the PPI network aimed to further understand the interactions of DEGs correlated with osteoporosis risk, consisting of 43 nodes and 218 edges (**Figure 2C**). The cytoHubba plugin additionally selected the top five genes from the PPI network (**Figure 2D**) as hub genes, including Assembly Factor for Spindle Microtubules (ASPM), Cyclin A2 (CCNA2), Cyclin B2 (CCNB2), TNF Receptor Superfamily Member 9 (CD137), Discs Large Homolog Associated Protein 5 (DLGAP5). The results of our study agree well with those of the previous study (17). Based on the above findings, we inferred that CD137 might be involved in the p53 and canonical Wnt/β-catenin signaling pathways and thereby influenced bone mass through regulation of marrow adipogenesis.

### Bone Loss and Bone Marrow Adipogenic Differentiation in Aged Mice

**Figure 3:**
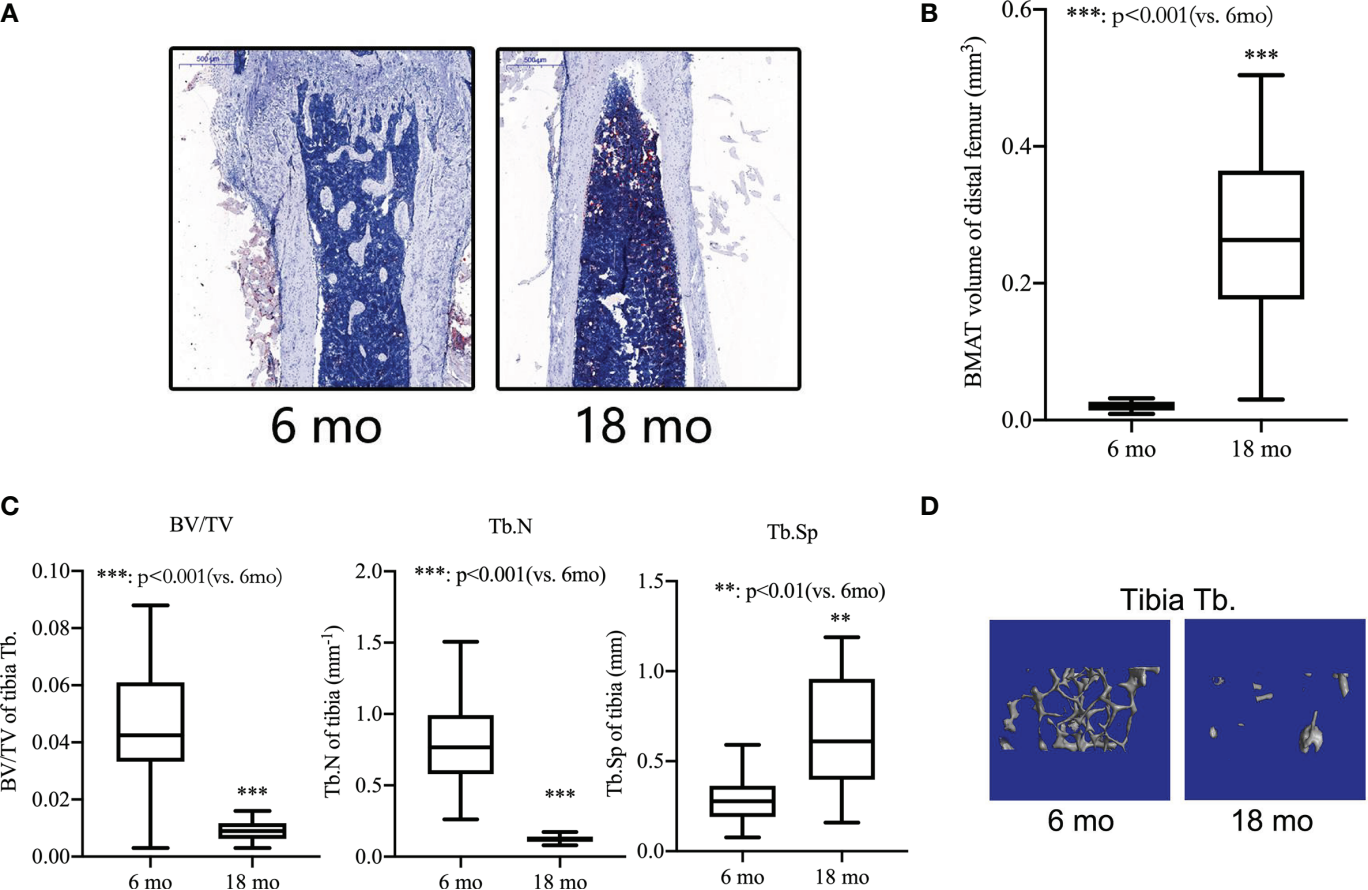
Bone loss and bone marrow adipogenic differentiation in aged mice. **(A)** Oil red-O stain of the medullary cavity of femur for intra-bone marrow in mice. **(B)** A morphometric analysis in the volume of bone marrow adipose tissue (BMAT). **(C)** Bone volume fraction (BV/TV), trabecular bone number (Tb.N) and trabecular bone space (Tb.Sp) of the proximal tibia from 6- and 18-month-old mice were determined by micro-CT. **(D)** Representative 3D reconstruction images of the proximal tibia Tb. All the data were obtained from three independent experiments. Data are shown as the means ± SEMs. **p < 0.01, ***p < 0.001, 18 mo vs. 6 mo.

During the first five to six months of a mouse’s life, they reach their maximum bone mass. Therefore, in the current study, senile mice were 8-18 months of age and 6-month-old mice were used as young mice. We separated the femurs and tibiae and collected them. An analysis of the bone microstructure was performed using micro-CT, and a measurement of the bone marrow adipose tissue volume was performed using Oil red-O staining. Compared with 6-month-old mice, Oil red O staining results showed that the adipose tissue of the medullary cavity of the femur in 18-month-old mice was significantly increased (**Figure 3A**). Morphometric data showed that the volume of adipose tissue in the femur intra-bone marrow increased more than 10 times in 18-month-old mice compared with 6-month-old mice (**Figure 3B**). Comparing 18-month-old mice with 6-month-old mice, there was a significant decrease in BV/TV, a decrease in Tb. N, and an increase in Tb.S in the proximal tibiae (**Figure 3C**). The same pattern was also seen in micro-CT 3D reconstruction images of the trabecular bone (**Figure 3D**). It has been shown that p53 Wnt/β-catenin signaling pathways can contribute to adipogenic differentiation of senile osteoporosis.

### Alleviation of Trabecular Bone Loss and Inhibition of Bone Marrow Adipogenic Differentiation in Aged Mice *via* Blocking of CD137 Signaling *In Vivo*

**Figure 4:**
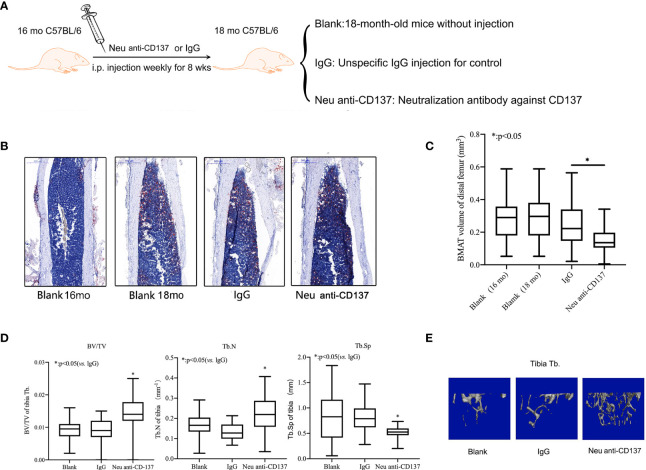
Alleviatation of trabecular bone loss and inhibition of bone marrow adipogenic differentiation in aged mice *via* blocking of CD137 signaling *in vivo*. **(A)** The 16-month-old C57BL/6 mice were intraperitoneally injected with neutralization antibody against CD137 once a week for 8 weeks. **(B)** Oil red-O stain of the medullary cavity of femur for intra-bone marrow in mice. **(C)** A morphometric analysis in the volume of bone marrow adipose tissue. **(D)** BV/TV, Tb.N, and Tb.Sp of the proximal tibia from 18-month-old mice with and without neutralization antibody against 4-1BB were determined by micro-CT. **(E)** Representative 3D reconstruction images of the proximal tibia Tb. All the data were obtained from three independent experiments. Data are shown as the means ± SEMs. *p < 0.05.

Weekly injections of neutralization antibody against CD137 were given intraperitoneally to sixteen-month-old mice (**Figure 4A**). Control recordings were made prior to the injection.The animals were euthanized after eight weeks to perform micro-CT scanning and Oil red O staining. There was no obvious difference between 16-month-old mice and 18-month-old mice on the basis of the Oil red O staining.Compared with IgG control, Oil red O staining results showed that the adipose tissue of the medullary cavity of the femur in mice administered neutralization antibody against CD137 was significantly decreased (**Figure 4B**). By morphometric analysis, we found a 30% decrease in adipose tissue volume in femur intra-bone marrows of mice that received neutralization antibodies against CD137 compared with mice that received IgG control antibodies (**Figure 4C**). In comparison with mice treated with IgG, neutralization antibodies against CD137 significantly increased BV/TV, a markedly increased Tb. N, and a marginally decreased Tb.Sp in the tibia (**Figure 4D**). Three-dimensional reconstruction images of micro-CT also showed the same pattern (**Figure 4E**). The results of these studies strongly suggest that the CD137 is important in the regulation of adipogenic capacity in senile osteoporosis.

## Discussion

Osteoporosis is a kind of systemic metabolic disease that is characterized by reduced degeneration of bone density, loss of bone mass, and degradation of bone microstructure (24). In the next decade, increasing numbers of people will suffer from osteoporosis (25). Osteoporosis is one of the most common chronic diseases which is accompanied by bone fragility, bone pain, and fracture (26). It has been considered as a serious worldwide health problem.

Molecular pathways and genes interact to cause senile osteoporosis, which is a complicated, multifactorial disease (27, 28). In the present study,241 DEGs were analyzed and screened from 3 gene expression profiles (GSE35956, GSE35957, and GSE35959) containing osteoporosis and normal tissue samples. Then, GO and KEGG analyses of DEGs were performed to understand the potential underlying pathways associated with osteoporosis. Cell component enrichment analysis showed that the DEGs significantly enriched in chromosome. The enrichment of GO assays is also in line with the literature (29). Based on the results of the KEGG pathway analysis, the Cellular senescence, p53 signaling pathway, and Cell cycle were significantly enriched pathway term. Osteoporosis were associated with cellular senescence in the current study, as predicted by the KEGG analysis. Previous studies have shown similar results (30). Cell cycle pathway also play an important role in osteoporosis progression (31). The p53 signaling pathway, in particular, appear to be more complex than initially anticipated.Previous studies showed that the p53 signal favored adipogenesis and lost osteogenic ability by the Wnt/ß-catenin signaling pathway (32). This supports our initial hypothesis. In order to explore the specific molecular mechanism, we constructed a PPI network and screened CD137, DLGAP5, ASPM, CCNA2 and CCNB2 as hub genes. Importantly, we found that these hub genes had been reported in previous studies. For example, CCNB2 as hub nodes plays an essential role in regulating bone remodeling of osteoporosis (33). CCNA2 also has important roles in initiating osteogenesis, adipogenesis, and chondrogenesis (34). In particular, CD137 not only has a high degree score in the PPI network but also are closely related to osteoclasts based on Feng’s study (35). CD137L and CD137 expressed on BMSCs promote the osteogenic differentiation through p53 Wnt/β-catenin signaling.What is more, we speculated that CD137 expression was up-regulated in BMSCs isolated of aged mice, affecting the osteogenic differentiation of BMSCs by the p53 Wnt/β-catenin pathway. Therefore, we eventually selected CD137 from potential hub genes to further develop this hypothesis. In rodent models of osteoporosis, decreased bone mineral density is closely correlated with bone marrow adipocytes’ accretion in the femur. What is more, the blockade of CD137 proved able to radically reverse age-related osteoporosis *in vivo*. These findings were consistent with our bioinformatics analysis researches.

Senescent cells accumulate with age and secrete senescence-associated molecules into the bone marrow microenvironment (36). Promoting adipogenic differentiation of BMSCs by these molecules was shown to inhibit osteogenic differentiation, which is responsible for the majority of osteoporotic bone loss (37). CD137 as one of the members of TNFRSF exists both as a membrane-bound and as a soluble cytosolic molecule. CD137’s ligand (CD137L) is a transmembrane polypeptide of the TNF ligand superfamily and exists in either a membrane-bound or a soluble form. CD137L is expressed on the surface of hematopoietic progenitor cells. The soluble form of CD137/CD137L seems to be produced by differential splicing. CD137 is an intracellular costimulatory receptor expressed by natural killer cells, T cells, and dendritic cells (26). Through reverse signaling, a combination of the two stimulates the activity of hematopoietic progenitor cells promoting their myeloid differentiation during aging functionally (26). Currently, osteoporosis is diagnosed based on the corresponding clinical symptoms and doctors’ experiences, mainly by following the radiographic changes in bone (38). However, clinical signs and symptoms are usually absent in the early stages of the disease. So, it has been proposed that biochemical markers involved in increased bone turnover can serve as potential indicators of the degree of bone loss. So, osteoporosis can be treated by monitoring bone turnover markers. Our study based on bioinformatics analysis found that the measurement of CD137/CD137L might provide a biomarker for identifying osteoporosis early and guiding osteoporosis treatment.

For interpretation of CD137 effects, it is important to understand its mechanism of action. According to bioinformatics analyses, CD137 plays a critical role in the p53 Wnt/β-catenin pathway that controls adipogenic differentiation. We tested this hypothesis by measuring the amount of trabecular bone loss in aged mice. As a result of the *in vivo* results, we have determined that young mice have a higher osteogenic differentiation potential than old mice by microarchitectural changes in trabecular bone. There also was a significant increase in adipogenic differentiation of aged mice compared with the control group. We find this interesting and in line with our previous work and hypothesis. Further experiments were conducted *in vivo* to validate the CD137 function. To block CD137 in BMSCs following CD137 signaling activation by CD137L, CD137 neutralizing antibody was employed. We found that CD137 enhanced osteogenic differentiation potential by inhibiting adipogenic differentiation in18-month-old mice. Combined with previous studies, CD137 might be a central factor in the impaired osteogenesis of aged mice. Trabecular bone loss in aged mice could be prevented by the CD137 antibody. In the past and present, there have been many efforts to treat inflammatory diseases with TNF-blocking antibodies such as adalimumab, etanercept, and golimumab (39). This study is the first of its kind to show that the CD137 antibody could alleviate trabecular bone loss by suppressing adipogenic differentiation.

**Figure 5:**
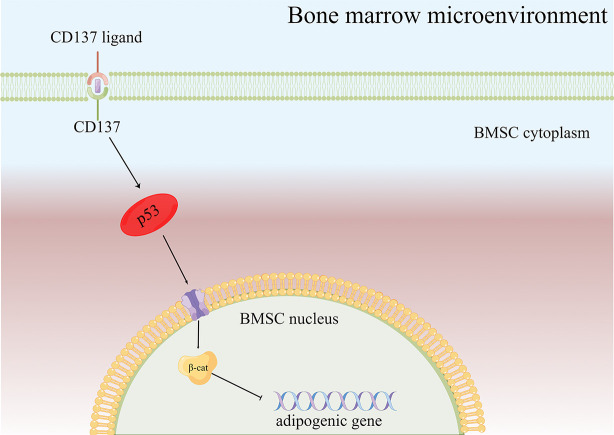
Diagram of the possible mechanism of bone loss *via* the CD137-p53 Wnt/β-catenin signaling pathways in aged mice. When CD137L binds to CD137 on BMSCs from aged mice, CD137 signaling is activated so that p53 Wnt/β-catenin signaling pathway is activated, then promotes the BMSC adipogenic differentiation potential of aged mice.

As a result of the present study, several major conclusions have been drawn. CD137 is the hub gene for osteoporosis based on the bioinformatics analysis. What’s more, we speculated that CD137 inhibited osteogenic differentiation by promoting adipogenic differentiation of BMSCs *via* the p53 Wnt/β-catenin pathway (**Figure 5**). Despite Oil red-O stain evidence, some of the specific mechanisms are still unclear and more research is required. Next, a further confirmation of the above conclusion can be found in the *in vivo* experiments by CD137 neutralization which could prevent trabecular bone loss in aged mice through suppression of adipogenic differentiation. However, it remains unclear the specific role CD137 deficiency plays in the p53 Wnt/β-catenin pathway. This mechanism needs to be verified *in vitro* through further experiments. Our research will contribute to our understanding not only of the pathogenesis of age-related bone loss but also to the identification of new targets for treating senile osteoporosis.

## Data Availability Statement

Publicly available datasets were analyzed in this study. This data can be found here: https://www.ncbi.nlm.nih.gov/geo/.

## Ethics Statement

The animal study was reviewed and approved by Ethics Review Committee of Shanghai Tongji Hospital. Affiliated Institution: Tongji Hospital affiliated to Tongji University.

## Author Contributions

JH extracted the data, finished the experiment, performed the statistical analysis, and drafted the paper. An investigation of literature and data validation was conducted by YW and HZ. DW and YZ both participated in the literature investigation and statistical analysis, as well as reviewing the manuscript. A version of the manuscript published in its final form has been reviewed and approved by all authors.

## Funding

Supported by the Fundamental Research Funds for the Central Universities (grant 22120210569)

## Conflict of Interest

The authors declare that the research was conducted in the absence of any commercial or financial relationships that could be construed as a potential conflict of interest.

## Publisher’s Note

All claims expressed in this article are solely those of the authors and do not necessarily represent those of their affiliated organizations, or those of the publisher, the editors and the reviewers. Any product that may be evaluated in this article, or claim that may be made by its manufacturer, is not guaranteed or endorsed by the publisher.

## References

[1] Clynes M Harvey N Curtis E Fuggle N Dennison E Cooper C. The Epidemiology of Osteoporosis. Br Med Bull (2020) 133(1):105–17. doi: 10.1093/bmb/ldaa005 PMC7115830 32282039

[2] Fischer V Haffner-Luntzer M. Interaction Between Bone and Immune Cells: Implications for Postmenopausal Osteoporosis. Semin Cell Dev Biol (2022) 123:14–21. doi: 10.1016/j.semcdb.2021.05.014 34024716

[3] Qadir A Liang S Wu Z Chen Z Hu L Qian A. Senile Osteoporosis: The Involvement of Differentiation and Senescence of Bone Marrow Stromal Cells. Int J Mol Sci (2020) 21(1):349. doi: 10.3390/ijms21010349 PMC6981793 31948061

[4] Duque G Demontiero O Troen B. Prevention and Treatment of Senile Osteoporosis and Hip Fractures. Minerva Med (2009) 100(1):79–94. 19277006

[5] Kanahori M Matsumoto Y Fujiwara T Kimura A Tsutsui T Arisumi S. Predictive Factors of Non-Treatment and Non-Persistence to Osteoporosis Medication After Fragility Hip Fractures at 3 Years After Discharge: A Multicentre, Prospective Cohort Study in the Northern Kyushu District of Japan. Arch Osteoporosis (2021) 16(1):132. doi: 10.1007/s11657-021-00988-5 34515859

[6] Nguyen C Schlesinger K James T James K Sah R Masuda K. Novel Magnetic Resonance Technique for Characterizing Mesoscale Structure of Trabecular Bone. R Soc Open Sci (2018) 5(8):180563. doi: 10.1098/rsos.180563 30225048 PMC6124118

[7] Weivoda M Chew C Monroe D Farr J Atkinson E Geske J. Identification of Osteoclast-Osteoblast Coupling Factors in Humans Reveals Links Between Bone and Energy Metabolism. Nat Commun (2020) 11(1):87. doi: 10.1038/s41467-019-14003-6 31911667 PMC6946812

[8] Xi Y Huang H Zhao Z Ma J Chen Y. Tissue Inhibitor of Metalloproteinase 1 Suppresses Growth and Differentiation of Osteoblasts and Differentiation of Osteoclasts by Targeting the Akt Pathway. Exp Cell Res (2020) 389(2):111930. doi: 10.1016/j.yexcr.2020.111930 32113948

[9] Che M Gong W Zhao Y Liu M. Long Noncoding Rna Hcg18 Inhibits the Differentiation of Human Bone Marrow-Derived Mesenchymal Stem Cells in Osteoporosis by Targeting Mir-30a-5p/Notch1 Axis. Mol Med (Cambridge Mass) (2020) 26(1):106. doi: 10.1186/s10020-020-00219-6 33176682 PMC7656763

[10] Yu C Peall I Pham S Okolicsanyi R Griffiths L Haupt L. Syndecan-1 Facilitates the Human Mesenchymal Stem Cell Osteo-Adipogenic Balance. Int J Mol Sci (2020) 21(11):3884. doi: 10.3390/ijms21113884 PMC7312587 32485953

[11] Yuan Z Zhang J Huang Y Zhang Y Liu W Wang G. Nrf2 Overexpression in Mesenchymal Stem Cells Induces Stem-Cell Marker Expression and Enhances Osteoblastic Differentiation. Biochem Biophys Res Commun (2017) 491(1):228–35. doi: 10.1016/j.bbrc.2017.07.083 28720497

[12] Li J Chen X Lu L Yu X. The Relationship Between Bone Marrow Adipose Tissue and Bone Metabolism in Postmenopausal Osteoporosis. Cytokine Growth Factor Rev (2020) 52:88–98. doi: 10.1016/j.cytogfr.2020.02.003 32081538

[13] Zhang S Feng P Mo G Li D Li Y Mo L. Icariin Influences Adipogenic Differentiation of Stem Cells Affected by Osteoblast-Osteoclast Co-Culture and Clinical Research Adipogenic. Biomed Pharmacother = Biomed Pharmacother (2017) 88:436–42. doi: 10.1016/j.biopha.2017.01.050 28122309

[14] Croft M Siegel R. Beyond Tnf: Tnf Superfamily Cytokines as Targets for the Treatment of Rheumatic Diseases. Nat Rev Rheumatol (2017) 13(4):217–33. doi: 10.1038/nrrheum.2017.22 PMC5486401 28275260

[15] Qiang R Zhao Z Tang L Wang Q Wang Y Huang Q. Identification of 5 Hub Genes Related to the Early Diagnosis, Tumour Stage, and Poor Outcomes of Hepatitis B Virus-Related Hepatocellular Carcinoma by Bioinformatics Analysis. Comput Math Methods Med (2021) 2021:9991255. doi: 10.1155/2021/9991255 34603487 PMC8483908

[16] Liu Y Cai G Chen P Jiang T Xia Z. Ube2e3 Regulates Cellular Senescence and Osteogenic Differentiation of Bmscs During Aging. PeerJ (2021) 9:e12253. doi: 10.7717/peerj.12253 34820159 PMC8606162

[17] Wan D Ai S Ouyang H Cheng L. Activation of 4-1bb Signaling in Bone Marrow Stromal Cells Triggers Bone Loss *Via* the P-38 Mapk-Dkk1 Axis in Aged Mice. Exp Mol Med (2021) 53(4):654–66. doi: 10.1038/s12276-021-00605-y PMC8102492 33859350

[18] Benisch P Schilling T Klein-Hitpass L Frey SP Seefried L Raaijmakers N. The Transcriptional Profile of Mesenchymal Stem Cell Populations in Primary Osteoporosis Is Distinct and Shows Overexpression of Osteogenic Inhibitors. PloS One (2012) 7(9):e45142. doi: 10.1371/journal.pone.0045142 23028809 PMC3454401

[19] Yu G Wang L Han Y He Q. Clusterprofiler: An R Package for Comparing Biological Themes Among Gene Clusters. Omics J Integr Biol (2012) 16(5):284–7. doi: 10.1089/omi.2011.0118 PMC3339379 22455463

[20] Walter W Sánchez-Cabo F Ricote M. Goplot: An R Package for Visually Combining Expression Data With Functional Analysis. Bioinformatics (2015) 31(17):2912–4. doi: 10.1093/bioinformatics/btv300 25964631

[21] Szklarczyk D Gable A Nastou K Lyon D Kirsch R Pyysalo S. The String Database in 2021: Customizable Protein-Protein Networks, and Functional Characterization of User-Uploaded Gene/Measurement Sets. Nucleic Acids Res (2021) 49:D605–D12. doi: 10.1093/nar/gkaa1074 PMC7779004 33237311

[22] Shannon P Markiel A Ozier O Baliga N Wang J Ramage D. Cytoscape: A Software Environment for Integrated Models of Biomolecular Interaction Networks. Genome Res (2003) 13(11):2498–504. doi: 10.1101/gr.1239303 PMC403769 14597658

[23] Bouxsein M Boyd S Christiansen B Guldberg R Jepsen K Müller R. Guidelines for Assessment of Bone Microstructure in Rodents Using Micro-Computed Tomography. J Bone Mineral Res (2010) 25(7):1468–86. doi: 10.1002/jbmr.141 20533309

[24] Ge W Jie J Yao J Li W Cheng Y Lu W. Advanced Glycation End Products Promote Osteoporosis by Inducing Ferroptosis in Osteoblasts. Mol Med Rep (2022) 25(4):140. doi: 10.3892/mmr.2022.12656 35211757 PMC8908347

[25] Adami G Fassio A Gatti D Viapiana O Benini C Danila M. Osteoporosis in 10 Years Time: A Glimpse Into the Future of Osteoporosis. Ther Adv Musculoskeletal Dis. doi: 10.1177/1759720x221083541 PMC8941690 35342458

[26] Anam A Insogna K. Update on Osteoporosis Screening and Management. Med Clinics North Am (2021) 105(6):1117–34. doi: 10.1016/j.mcna.2021.05.016 34688418

[27] Saad F. Novel Insights Into the Complex Architecture of Osteoporosis Molecular Genetics. Ann New York Acad Sci (2020) 1462(1):37–52. doi: 10.1111/nyas.14231 31556133

[28] Tobias J Duncan E Kague E Hammond C Gregson C Bassett D. Opportunities and Challenges in Functional Genomics Research in Osteoporosis: Report From a Workshop Held by the Causes Working Group of the Osteoporosis and Bone Research Academy of the Royal Osteoporosis Society on October 5th 2020. Front Endocrinol (2020) 11:630875. doi: 10.3389/fendo.2020.630875 PMC7917291 33658983

[29] Yang H Zhang B Zhu J Liu D Guan F He X. 4q22.1 Contributes to Bone Mineral Density and Osteoporosis Susceptibility in Postmenopausal Women of Chinese Han Population. PloS One (2013) 8(11):e80165. doi: 10.1371/journal.pone.0080165 24278256 PMC3836996

[30] Farr J Rowsey J Eckhardt B Thicke B Fraser D Tchkonia T. Independent Roles of Estrogen Deficiency and Cellular Senescence in the Pathogenesis of Osteoporosis: Evidence in Young Adult Mice and Older Humans. J Bone Mineral Res (2019) 34(8):1407–18. doi: 10.1002/jbmr.3729 PMC6697189 30913313

[31] He F Luo S Liu S Wan S Li J Chen J. Zanthoxylum Bungeanum Seed Oil Inhibits Rankl-Induced Osteoclastogenesis by Suppressing Erk/C-Jun/Nfatc1 Pathway and Regulating Cell Cycle Arrest in Raw264.7 Cells. J Ethnopharmacol (2022) 289:115094. doi: 10.1016/j.jep.2022.115094 35149133

[32] Zhou X Beilter A Xu Z Gao R Xiong S Paulucci-Holthauzen A. Wnt/ß-Catenin-Mediated P53 Suppression Is Indispensable for Osteogenesis of Mesenchymal Progenitor Cells. Cell Death Dis (2021) 12(6):521. doi: 10.1038/s41419-021-03758-w 34021120 PMC8139956

[33] Wang C Yu T Tan L Cheng J. Bioinformatics Analysis of Gene Expression Profile in Callus Tissues of Osteoporotic Phenotype Mice Induced by Osteoblast-Specific Krm2 Overexpression. Int J Rheumatic Dis (2016) 19(12):1263–71. doi: 10.1111/1756-185x.12840 26929007

[34] Liu F Dong J Zhang P Zhou D Zhang Q. Transcriptome Sequencing Reveals Key Genes in Three Early Phases of Osteogenic, Adipogenic, and Chondrogenic Differentiation of Bone Marrow Mesenchymal Stem Cells in Rats. Front Mol Biosci (2021) 8:782054. doi: 10.3389/fmolb.2021.782054 35223983 PMC8873985

[35] Feng W He M Jiang X Liu H Xie T Qin Z. Single-Cell Rna Sequencing Reveals the Migration of Osteoclasts in Giant Cell Tumor of Bone. Front Oncol (2021) 11:715552. doi: 10.3389/fonc.2021.715552 34504794 PMC8421549

[36] Ovadya Y Landsberger T Leins H Vadai E Gal H Biran A. Impaired Immune Surveillance Accelerates Accumulation of Senescent Cells and Aging. Nat Commun (2018) 9(1):5435. doi: 10.1038/s41467-018-07825-3 30575733 PMC6303397

[37] Shuai Y Yang R Mu R Yu Y Rong L Jin L. Mir-199a-3p Mediates the Adipogenic Differentiation of Bone Marrow-Derived Mesenchymal Stem Cells by Regulating Kdm6a/Wnt Signaling. Life Sci (2019) 220:84–91. doi: 10.1016/j.lfs.2019.01.051 30710639

[38] Curry S Krist A Owens D Barry M Caughey A Davidson K. Screening for Osteoporosis to Prevent Fractures: Us Preventive Services Task Force Recommendation Statement. JAMA (2018) 319(24):2521–31. doi: 10.1001/jama.2018.7498 29946735

[39] Cheng J Zhang J Wu Z Sun X. Corrigendum To: Inferring Microenvironmental Regulation of Gene Expression From Single-Cell Rna Sequencing Data Using Scmlnet With an Application to Covid-19. Briefings Bioinf (2021) 22(2):1511–2. doi: 10.1093/bib/bbab015 PMC7799217 33341869

